# Chromatin Computation

**DOI:** 10.1371/journal.pone.0035703

**Published:** 2012-05-02

**Authors:** Barbara Bryant

**Affiliations:** Bioinformatics Department, Constellation Pharmaceuticals, Cambridge, Massachusetts, United States of America; Queen Mary, University of London, United Kingdom

## Abstract

In living cells, DNA is packaged along with protein and RNA into chromatin. Chemical modifications to nucleotides and histone proteins are added, removed and recognized by multi-functional molecular complexes. Here I define a new computational model, in which chromatin modifications are information units that can be written onto a one-dimensional string of nucleosomes, analogous to the symbols written onto cells of a Turing machine tape, and chromatin-modifying complexes are modeled as read-write rules that operate on a finite set of adjacent nucleosomes. I illustrate the use of this “chromatin computer” to solve an instance of the Hamiltonian path problem. I prove that chromatin computers are computationally universal – and therefore more powerful than the logic circuits often used to model transcription factor control of gene expression. Features of biological chromatin provide a rich instruction set for efficient computation of nontrivial algorithms in biological time scales. Modeling chromatin as a computer shifts how we think about chromatin function, suggests new approaches to medical intervention, and lays the groundwork for the engineering of a new class of biological computing machines.

## Introduction

### Computation as a metaphor for cellular function

Computer programs and logic circuits have often been used as metaphors for the function of cells [1], [2]. A cell may be considered to be executing a program not unlike that of a computer. Given inputs such as the cellular environment, the cell “computes” outputs and behaviors such as secreted factors, shape changes, and cell division. One might consider a multi-cellular organism to have been “computed” from a single cell. Evolution itself can be considered a computation, and has inspired a class of computer algorithms conceived by Turing in 1948 [3], and variously called genetic algorithms, evolutionary programming or evolution strategies [4].

A computer implements a set of rules that operate on memory. A formal definition of computation was invented by Turing, whose theoretical machine could read and write symbols on an infinitely long tape according to a finite set of rules [5]. Church's thesis states that every algorithm can be computed by a Turing machine – including algorithms that cannot be computed by finite state automata or logic circuits. Any model of computation (system of rules operating on data) that can simulate a Turing machine is also, therefore, computationally universal.

Several authors have shown that DNA can be used to simulate a Turing machine [6], [7], [8], [9]. In each of these examples, the Turing tape is mapped to DNA, and the Turing rules are mapped to DNA operations like reading (using DNA base pairing), cutting (using restriction enzymes that recognize and cut at a specific DNA sequence), and reconnecting (using DNA ligation at overhanging complementary DNA sequences and/or DNA polymerase). To simulate a Turing machine, the read/write head location and machine state are encoded using a special state symbol (sequence) at one specific location in the DNA. The execution of a rule involves using DNA base pairing to read the current state and symbol, and then cutting out old and inserting new DNA to move the head or write a new symbol. While these and other biologically-based universal DNA computers are interesting theoretically, they do not model what really happens in a cell. Nor are they practical for real problems: the lab operations are time consuming and error-prone, and they are not easy to program.

In 1994, Adleman made headlines with a DNA computer that solved an instance of the NP-complete Hamiltonian path problem [10]. Following this initial success, other interesting problems were shown to be solvable with actual biochemical manipulations [11], [12], [13], [14]. While these examples show that DNA computers can solve specific instances of problems, it is harder to cope with more general problems such as multiplying two arbitrarily large integers. These approaches do not provide an easy way to write general-purpose programs; the solutions tend to be closely tailored to both the computational model and the particular problem. The execution of the program is time-consuming, as multiple laboratory steps are required. The solutions tend to take advantage of massive parallelism to try many different solutions to find one that works; it is much harder, if not impossible, to program such systems to deterministically explore a search tree.

Other forms of biomolecular computation include chemical kinetics, membrane computing, pi-calculus and the blob model [15], [16], [17], [18], [19], [20]. Some of these were initially developed to study systems of interacting computations, and were later applied to model biomolecular systems. While inspired by real biomolecular behavior, these approaches are, so far, more synthetic than analytic: they are programmable, but they are either hard to program, not practical to implement, or stray from modeling real biology. Some approaches rely on precise quantitation of the number of molecules, and require engineering of one-way reactions that can be chained and networked in arbitrary graph topologies.

Transcription factor control over gene expression is often expressed as a logic circuit: a combination of AND, OR and NOT operations on Boolean values – consider, for example, the lac repressor [21]. Diagrams to describe biological gene networks have become common, often using the visual vocabulary of digital logic circuits [22]. Real implementations of basic circuit elements in *E. coli* include an artificial oscillating network [23], and a toggle switch [24]; design principles of more complex circuits have been laid out [25], [26], [27]. While these are useful models of biological systems, they are not universal computational systems, and cannot be programmed to solve problems of arbitrary complexity.

Thus, existing models of biological computation are either powerful computationally but impractical, or not universal – and in neither case are they easy to program.

### The histone code

In living cells, DNA is packaged along with protein and RNA into chromatin. DNA methylation has long been associated with control, and particularly repression, of gene transcription [28], [29]. In 2000, the term “histone code” was coined to capture the idea that post-translational modifications on histone proteins might have specific functions, and be read, erased and written by specific modifiers and effectors [30], [31]. A related term, “epigenetic code”, emphasized the idea that these molecular modifications were stable enough to encode information, apart from DNA sequence, that could be transmitted, in some cases, from parent to daughter cells [32]. In the years since the proposal of this paradigm, biologists have indeed elucidated the specific read/erase/write functionality of many histone modifying protein domains [33], [34], [35].

Chromatin-reading and -writing proteins operate as components of molecular complexes that read and write multiple marks in a combinatorial fashion. These complexes often include transcription factors that recognize specific DNA sequences, as well as effector units that carry out gene transcription or other functions, and scaffolding proteins or RNA to bring the right components together into the complex. The phenomenon of engaging multiple marks at once is often referred to as “multivalency” of chromatin modifiers, or “cross-talk” between combinatorial marks [32], [36], [37], [38]. Reading units within one complex may target marks within one histone, in different histones in the same nucleosome, or even across multiple nucleosomes [39]. The same protein may take part in different complexes depending on which subunits are currently available. Thus, a chromatin-modifying complex can be thought of as a read-write rule with the following form: “Find a nucleosome adjacent to DNA sequence AGCCAT; if it is marked with H3K4me3 and H3K27ac, and the DNA is not methylated, then mark the next nucleosome with H3K4me3 and start transcription of a gene.”

These rules may operate sequentially on chromatin at a particular location. For example, in animal development, the DNA methylation pattern is erased in the early embryo [40], a new pattern established by the time of implantation, and further altered over the course of somatic development. Gametogenesis also involves coordinated erasing and rewriting of DNA methylation. These developments are carried out in a series of steps involving chromatin modification read-write rules implemented by complexes [41].

### An idealized model of chromatin

Here I present a new computational system, in which chromatin is the writable memory and chemical modifications are the written symbols. Read-write rules model the molecular complexes that recognize and place specific combinations of DNA and histone modifications. The formalism can be easily “programmed” to solve problems such as the NP-complete Hamiltonian path problem, either by the same massively parallel guess-and-check approach of Adleman, or by a more deterministic algorithm that traverses the search tree, with backtracking.

I prove that chromatin computers are Turing-complete by using one to simulate a Turing machine. The mapping to a Turing machine is not forced, but uses components whose complexity is no greater than that of biological chromatin. I implement a script to simulate execution of chromatin computer programs. I show that biological chromatin has many features that provide computational efficiency, such as parallelism, nondeterminism, addressable memory, modification of the program during computation, and topological shortcuts. The chromatin computer formalism is thus both a natural model of biological chromatin, and a powerful language in which to write computer programs.

## Results

### Formal definition of a chromatin computer

A chromatin computer (CC) has a set of read-write rules that operate non-deterministically on **chromatin**, which is an infinite string of **nucleosomes**, analogous to a Turing tape. Each nucleosome consists of *k* adjacent chromatin **positions**. Each position contains a chromatin **mark** drawn from an alphabet of finite size *m*. The marked chromatin defines the CC's **configuration** at any point in the computation.

A CC is defined by the tuple 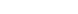 as follows:

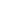
 is a finite, non-empty set of possible chromatin marks, of size *m*.

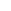
 (for “blank”) represents the absence of any chromatin mark, and is an element of 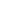. (We will call 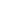 a mark even though it means the absence of any actual chromatin mark.)

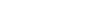
 is a transition function, or set of read-write rules. Each rule reads the modifications at 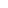 adjacent nucleosomes, and then writes updated marks at those same positions. 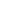 is the set 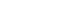 , and 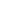 is the set 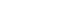 .

The CC operates non-deterministically on an input chromatin configuration, which is marked everywhere by B, except for a finite number of nucleosomes which may have other marks. At each step, the read portion of zero or more rules will match at various locations along the chromatin tape. One matching rule is selected at random and applied to update the modifications at that location. If no rule matches at any location on the chromatin, then the CC halts.

The left hand side of each rule is a read specification for all or some of the marks at *n* adjacent nucleosomes. The special symbol * can be used in the reading specification to match any mark. (This serves both to more closely model real chromatin reading complexes, and to simplify the writing of the rules.) The write specification of a rule may employ the special “no-change” symbol –.

**Figure 1:**
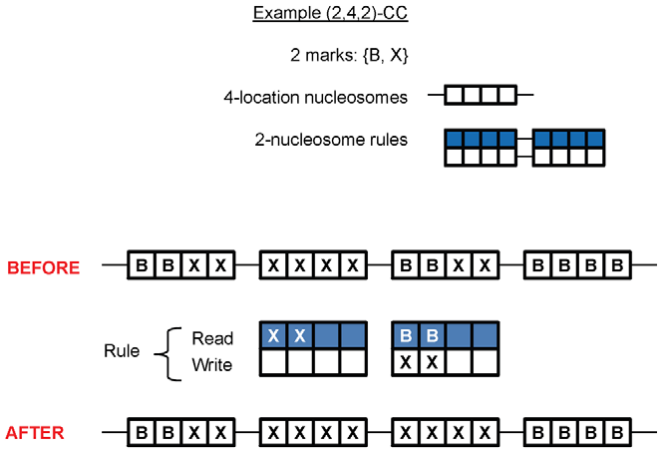
Example of the operation of a chromatin computer rule. This diagram illustrates the operation of the rule XX** BB** 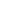 ---- XX--. The chromatin tape is composed of nucleosomes having four writable locations. Each location can be marked with the symbol B or X. Rules in this CC operate on two adjacent nucleosomes. In the illustration of the read portion of the rule, matching to any symbol (*) is shown with an empty. An empty square in the write portion of the rule leaves the current symbol unchanged (−).

Chromatin consisting of nucleosomes that each have *k* mark positions is referred to as *k*-**chromatin**. A CC that operates on *k*-chromatin, with rules addressing *n* adjacent nucleosomes, and reading and writing marks from an alphabet of size *m*, is referred to as an (*m,k,n*)**-CC**. Figure 1 illustrates the application of the (2,4,2)-CC rule XX** BB** 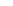 ---- XX--.

### Solving a directed Hamiltonian path problem with a chromatin computer

**Figure 2:**
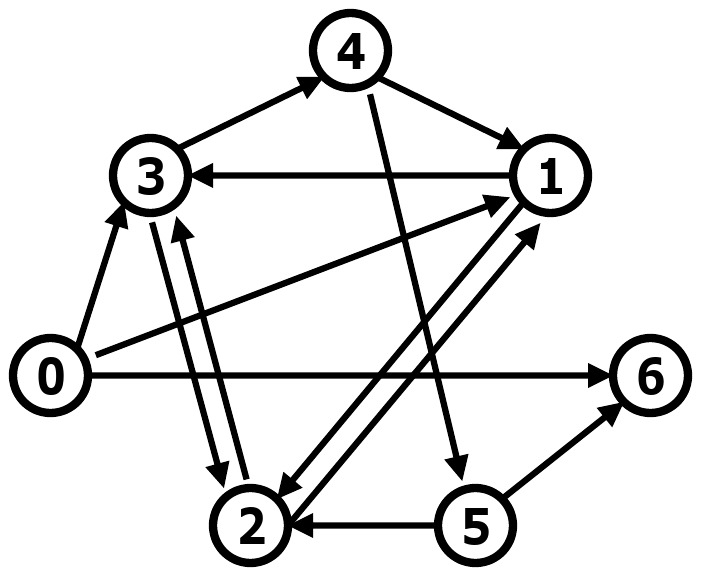
Hamiltonian path problem. Figure from Adleman 1994 (5). In the pictured directed graph, there is a unique Hamiltonian path from vertex 0 to vertex 6: 0
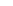
1
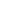
2
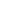
3
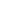
4
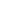
5
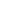
6.

In 1994 Adleman created a DNA-based solution to an instance of the Hamiltonian path problem. Let us tackle the same problem, shown in Figure 2, to illustrate use of a chromatin computer.

The Hamiltonian path problem asks whether there exists a path in a directed graph from the input vertex to the output vertex, visiting each of the other vertices exactly once. Adleman synthesized 20-mer oligonucleotides representing the vertices and edges in the graph. The sequence of an edge's 20-mer was complementary to the appropriate halves of its two vertices' 20-mers. These 20-mers were mixed together and ligated, resulting in double-stranded DNA representing valid paths through the graph. Further sizing and affinity purification steps ensured that each node was represented once and only once in the soup of path-representing oligonucleotides. The sequence of nodes in the correct path was determined using PCR and running the products on a gel. The number of starting 20-mers was large enough that production of the correct path was highly likely.

**Figure 3:**
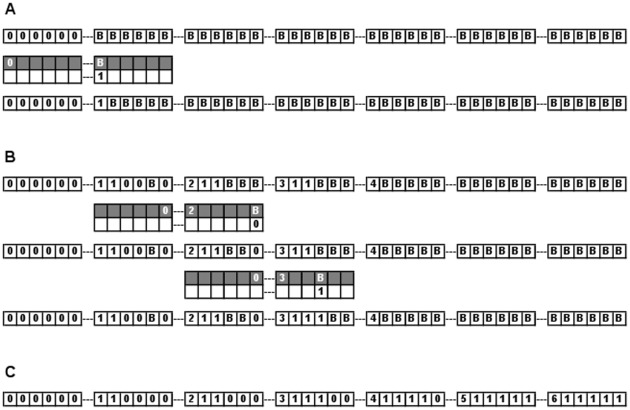
Operation of a chromatin computer solving the Hamiltonian Path Problem. (A) Application of a rule to the starting configuration. The chromatin tape is shown as a set of 7 nucleosomes, each with 6 writable positions. The top row shows the initial tape configuration; the bottom row shows the configuration after the application of the rule 0***** B***** 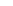 ------ 1-----. The leftmost position in each nucleosomes maps to a numbered vertex in the graph. The remaining 5 positions are used to determine whether each node appears exactly once in the path. This rule extends the path from 0 to 1. (B) Two path-checking rules operating sequentially on an intermediate configuration in the computation. (C) The final chromatin configuration showing the successful solution.

Our first implementation of a solution to this problem using a chromatin computer will employ a similar guess-and-check approach, by randomly constructing many paths of up to 7 nodes, and signaling success only for a path meeting the requirements. The solution uses 6-chromatin: each nucleosome has six read/write positions. Each rule looks at two adjacent nucleosomes, and there are 10 possible marks, so the CC is a (10,6,2)-CC. One position in each nucleosome represents the vertex number, and the remaining five are used to check that the path contains one and only one visit to each vertex. Figure 3 illustrates CC configurations along the way to the correct solution.

Additional explanation, the full rule set and a perl script to simulate the chromatin computer are provided in Text S1. A second variant solution takes a single run of the computer, using backtracking to randomly explore paths in the graph. A third variant uses an insulating nucleosome to make the search more efficient. Thus we see that it is possible to write programs that can trade off computational space for time – it is possible to solve the Hamiltonian path problem not just by trying every possible path in parallel, but also by backtracking in an orderly fashion to completely search all possible paths, in a single deterministic computation. This is not possible in the DNA-based computational model. Moreover, no intermediate lab operations, such as cooling to anneal, or running gels to filter solutions to the correct size, are required; once the rules are mixed with the starting chromatin tape, the computation proceeds to completion on its own.

### Chromatin computers are Turing complete

A Turing machine is defined by its finite set of rules; each rule specifies a mapping from a symbol and state to a new symbol, a new state, and a movement left or right along the memory tape. A configuration of a Turing machine comprises a machine state, a location of a read/write head on the infinite memory tape, and the contents of the tape. Initially, the tape is blank except for symbols written at a finite number of cells. At each step in the computation, the rule corresponding to the symbol at the current tape cell and the current machine state is applied, and specifies the writing of a new symbol at the current tape cell, a new machine state, and a movement left or right along the tape. If no rule applies, the machine halts.

**Figure 4:**
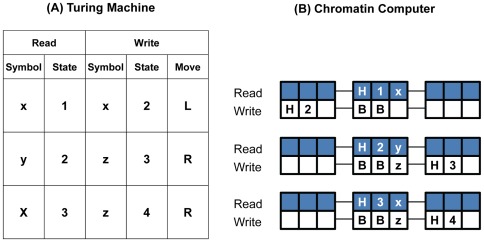
Mapping from a Turing machine to a chromatin computer. (A) Turing machine finite state machine with three rules that rewrite the string “xy” to “zz”. (B) The corresponding chromatin computer. The first position in each 3-position nucleosome corresponds to the location of the Turing read/write head. The second position corresponds to the state of the Turing machine. The third position corresponds to a cell on the Turing tape.

To prove that a chromatin computer can simulate a Turing machine, I define a reversible mapping from any Turing machine to a chromatin computer, and from each Turing configuration to a chromatin configuration. I then show by induction that running the chromatin computer results in a chromatin configuration that maps back to the Turing configuration that would have been achieved by running the Turing machine, and that the chromatin computer halts whenever the Turing machine halts. The trick to the mapping is to transform each Turing tape cell to a nucleosome, with extra nucleosome positions to store the current state and the current location of the read/write head. Moving left or right along the Turing tape is accomplished on the chromatin by moving these state and head-location marks to adjacent nucleosomes. Figure 4 illustrates the mapping of 3 rules from a Turing machine to the corresponding chromatin rules. The full proof is provided in the methods section.

### Biological chromatin is a massively parallel, random access, self-modifying stored procedure computer

Computer scientists have developed many models of computation that are far more efficient and easier to program than the basic single-head non-deterministic Turing machine. These variations are no more powerful than a Turing machine from a computability standpoint. Non-deterministic Turing machines allow more than one rule with the same left-hand (read) side, and therefore many possible computational paths. Parallel Turing machines have multiple read-write heads, all operating simultaneously. Multi-tape Turing machines have several tapes and corresponding read/write heads. Random access machines allow incrementing and decrementing values in addressable registers. Indirect addressing allows a memory address to be operated on as data. Stored procedure models allow the program itself to be specified as input. Modern computer programming languages are no more capable than a Turing machine of solving a problem, but they can be programmed far more easily, and use fewer computational steps.

Just as real computer languages are more practical than Turing machines, biological chromatin implements many efficiencies either available in our initial CC model, or easily added to it. These efficiencies are powerful; they are exploited by living cells and make programming a simulated CC much easier. Some are familiar concepts from computer science; others are less familiar and quite interesting as computational tricks.

#### Non-determinism

The CC model is nondeterministic, although any particular CC may, by virtue of its rule set, be deterministic. The CC formulation encourages us to ask the question of whether, in a cell, more than one expressed chromatin-modifying complex could match and operate at a given location in a particular configuration of biological chromatin, or whether the design is deterministic. In order to implement consistently repeatable behavior, it seems likely that biological computation has constrained non-determinism in the sense that a given starting chromatin configuration with a given rule set is likely to evolve in a fairly consistent manner upon repeated runs, even if the details of the precise order of rule application at different locations may change from one run to another. This will be an interesting area for future work in modeling biological chromatin modifying complexes.

#### Multiple copies of chromatin-modifying complexes; parallel computation

There are many copies of chromatin-modifying complexes present in the cell, and they operate in parallel throughout the genome. Parallel rule application is readily handled by modifying the definition of a CC to allow not just one, but any number of non-overlapping, matching rules to be applied at each step. To capture the number of physical copies of a complex, the definition of *R* can be augmented to *R x Z* (where *Z* is the set of non-negative integers), allowing a mapping from each rule to the number of copies that exist. At each step, a restriction can be placed that a rule can match any number of valid chromatin tape locations, up to this integer upper limit. In a further variation of the computer, the likelihood of a rule matching at a location can be made proportional to the number of available copies of that rule. Parallel computation results in a huge speed-up over serial computation. In a yeast cell, there are 30,000 copies of just one nuclear complex component, RNA polymerase II [42]. This is a reasonable under-estimate for the number of chromatin-modifying complexes present in a nucleus.

#### Procession of complexes along chromatin

Some chromatin-modifying complexes, such as those containing RNA polymerase, are known to operate sequentially along the genome. While this can be programmed in our current CC model by having a special mark representing the current location of a rule, it can also be efficiently handled by augmenting the model to allow the right-hand side of a rule to have an additional field for movement: one of {left, right, disengage}. “Disengage” indicates that the rule would not subsequently be applied to the adjacent chromatin position; “left” and “right” indicate an immediate application to the neighboring position. With this notion of walking along the chromatin tape, we have resurrected the left and right movement of the Turing head in the Turing machine system.

#### Looping

Chromatin is known to form loops, allowing fairly distant regions along a chromosome to come into physical contact [43], [44], resulting in a topology beyond one dimension with connections that can change over time. This brings transcription factors bound at enhancers into close proximity with the promoter of the genes whose expression they control. It also seems to segregate segments of chromatin into nuclear compartments that have different chromatin state. Often many transcription complexes come together into a transcription factory [45]. The CC model can be extended to allow reading across these contact regions of loops, effectively spanning long linear distances along the chromatin tape. One approach to this model extension builds on the rule processing of the previous section, in which a rule moves left or right along the chromatin tape upon completion of a step. To model looping, we can define a further extension in which a rule, when it first binds, binds adjacent nucleosomes. But at each rule application, the each nucleosome component in the rule can be stepped left or right along the chromatin tape (constrained, however, to retain the relative order within the rule). In effect, this allows the creation of a loop, where the rule is attached to both ends of the loop using different rule components. An application of this capability could be to hold onto one chromatin location while walking along the tape to find another mark indicating the end of this loop. There is biological precedent for such a mechanism: the lagging strand of DNA replication fork creates loops called Okazaki fragments; one component of the DNA polymerase complex is attached to a fixed location on the DNA, while another component walks along the DNA, generating a growing loop.

#### Transcription factors that bind DNA motifs

Transcription factors are proteins that bind specific DNA sequences and carry out actions including chromatin modification, recruitment of additional proteins, and activation or suppression of gene expression. Transcription factors are easily modeled in our existing CC formalism as rule components that read marks corresponding to the DNA sequence co-localized with a nucleosome. The chromatin tape is initialized at each nucleosome with read-only marks representing the DNA sequence. Transcription factor binding site recognition is analogous to a “GOTO” instruction referencing an addressable memory cell in a random-access computer – a huge efficiency in programming. (One difference is that, while rare, the transcription factor binding sequence usually occurs multiple times in a genome, retaining an element of parallelism.)

Importantly, transcription factors alone would be insufficient to implement our mapping to a Turing machine, because of the lack of the ability to write to the chromatin tape.

A transcription factor of particular interest is CTCF. CTCF is known to have a role both in looping and as an insulator stopping the spread of marks along chromatin [46]. The insulator function is a handy programming tool for the CC to set spatial boundaries on computations; in fact, an insulating mark is used in one of our implementations of the Hamiltonian Path solution. It is straightforwardly implemented as a chromatin mark in the basic CC model.

#### Nucleosome remodeling

Nucleosome remodelers remove, replace and shift histone octamers along the genome. Removal and replacement can easily be modeled with our existing CC as rules that simply change the marks on a CC nucleosome. If we are modeling DNA sequence for transcription factor binding, then nucleosome shifting relative to that sequence can be modeled in a number of ways. For example, a straightforward modification of the CC model would accommodate a second, read-only, tape for the DNA sequence with an alignment to the nucleosome tape. Rule functionality can be expanded to allow local changes in the alignment.

#### Gene expression

The Turing machine does not formally produce output beyond halting, in a final state. In practical applications of Turing machines or their variants, the symbols on the tape are usually read after the computation halts, providing useful output from the computation. In a synthetic implementation of a CC, it might be useful to read the chromatin marks after a computation has been carried out, but another readout can be gene “expression” implemented by a rule that reports the occurrence of an expression event from a particular chromatin tape location. The CC formalism is easily augmented to accommodate gene expression: the right hand side of each rule includes an output symbol corresponding to the genomic location of the chromatin.

#### Signaling

Cell signaling changes chromatin state. A typical signaling cascade starts with binding of an extracellular ligand to a surface receptor, then transfers information via phosphorylation of a cascade of kinases; ultimately a transcription factor binds DNA and recruits additional complex proteins to effect a change in chromatin state and gene expression. In the CC model, this corresponds to a change of the program (or rule set), adding a rule involving the transcription factor complex.

#### Feedback: expression of chromatin-modifying complex genes

Stored procedure computers, or universal Turing machines, store the programming instructions on the input tape instead of hard-coding them into the rules. The hard-coded rules interpret and execute the instructions (the “software”) on the tape. Since chromatin computers can simulate any Turing machine, they can simulate universal Turing machines. But the gene expression augmentation to the CC formalism provides a natural biological model for stored programs. The CC rules represent chromatin modifying complexes, which are collections of expressed gene products. Then biological CC rules are, indeed, written in the input chromatin: the gene products self-organize into new rules – and these rules in turn change chromatin state and gene expression. Thus biological chromatin is not only a stored procedure computer, but a self-modifying stored procedure computer.

#### Replication

DNA, and some of the associated chromatin marks, is replicated when cells divide. Copying of chromatin state is readily modeled in the CC formalism; however, a more convenient addition to the formalism is creation of a copy of the current tape, analogous to a multi-tape Turing machine.

#### Summary

Biological chromatin plays a complex role in cell biology. Many of the features of chromatin-interacting factors can be modeled as efficiency-gaining instructions in the CC programming toolbox. I have mentioned some of them above; there are more. None of these features invalidate the powerful Turing completeness result that rests on the model of a linear array of writable nucleosome positions operated on by a finite rule set.

Table 1 summarizes the mapping from computational features to their biological counterparts under the CC model. The core power of the CC model lies in the combination of a finite read-write rule set with a large writeable memory.

**Table 1:** Biological equivalents for computational concepts in the CC model.

Computational concept	Biological equivalent
Writeable memory	Chromatin with chemical modifications
Read-write rules	Chromatin-modifying complex (CMC)
Parallel computer	Multiple copies of CMCs
Non-determinism	Different CMCs that read the same chromatin configuration
Addressable memory	Transcription factors binding specific DNA sequences
Output	Gene expression or chromatin configuration
Stored procedures	Genes coding for CMC components
Self-modifying code	Changing expression of genes coding for CMC components

### Memory size, rule set and clock speed of biological chromatin computers

To ask whether biological chromatin has the memory, rule set and speed capacity to carry out interesting computations, we can start from known biology. In Text S1, I calculate that each human cell contains at least 80 megabytes of writeable chromatin – a plentiful amount compared to, say, the 150,000 bytes of onboard memory in the Apollo mission that got astronauts to the moon.

How rich are the programs that operate on that memory? Text S1 lists 39 known nuclear complexes, each with multiple read and write functions (provided by proteins, each of which may itself have multiple read, write and connector domains). Because of the combinatorial construction of these complexes, including not only read and write components but also connector scaffolding proteins, it is likely that there are at least hundreds of these read/write complexes implemented in living cells. And that does not even take into consideration DNA-reading transcription factors – of which there are hundreds [47]. Each transcription factor plays a reader role in one or more effector complexes.

RNA polymerase II is a protein complex that transcribes DNA. Associated with polymerase function are factors that mark histones – for example, methylation of H3K4 and H3K36. Let us therefore take RNA polymerase II as one example of a chromatin-modifying complex and consider the rate at which it operates in the cell. One complex transcribes up to 90 nucleotides per second [48]; let's call it 50 nt/s. If there is a nucleosome every 200 nt, Pol II therefore proceeds along the chromatin at a rate of 0.5 nucleosomes/s. With 30,000 Pol II complexes in the cell [42], and perhaps 20,000 of them engaged, we have a rate of 10,000 nucleosomes processed each second, or 10,000 Hz. This represents an under-estimate for computation rate in a cell.

An alternate calculation starts from the assumption that an average read/write operation might take 1 second, and that at any point in time 1% of the cell's 10,000,000 nucleosomes might be engaged at position 1 of a read/write complex. This gives us an estimate of 1,000,000 operations per second, or 1 MHz.

Our lower estimate of the compute power of biological chromatin, then, gives us hundreds of different rules operating on at least 80 megabytes of memory at a minimum of 10,000 operations per second. While a living cell may not use this capability to its fullest, it represents enormous capacity for information processing on biological time scales.

## Discussion

Each human cell contains at least 80 megabytes of writeable chromatin (see Text S1 for the calculation). What computations might real chromatin carry out using this memory? Some programs' function is known. Some marks spread along a chromosome until they reach insulators. Histone modifications are important in development; for example, they mark “poised” promoters in pluripotent cells, and descendent cells have one or the other of those modifications. Chromatin can “burn in” repressive marks, making them more permanent over time [41]. Chromatin modifications are involved in exon selection, in tagging enhancers differently in different cell types [49], and in transcriptional pausing control [50]. Choreographed chromatin modifications play an important role in the highly ordered, stage-specific V(D)J combinatorial rearrangement of immune system antigen- and self-recognition proteins [51]. We are beginning to tease out the individual steps in these computations, with experimental work ranging from structural biology to designer histones to RNAi and chemical inhibition of modification-altering enzymes, as well as protein-protein interaction and genome-wide occupancy assays.

Modeling chromatin as a computer suggests a number of lines of inquiry. DNA methylation is erased in the early embryo; does this serve a similar function to rebooting a computer – resetting memory to enable restarting of programs? Genes with variable expression tend to have nucleosome-free regions (NFRs) further upstream of their transcription start site than constitutively expressed genes [52]. Might this be to “leave room” for more computation along the nucleosome tape? The variable-expression genes also have more histone turnover, another potential sign of active computation. How can we achieve robustness of the system in the face of possible “bugs” introduced by mutations in the rules? What are the characteristics of a robust symbol (modification) set for computation? How do the computational programs evolve? Are there characteristics of the rule sets that make them more robust in an evolutionary sense? Could we evolve a useful chromatin computer program in silico?

With the intensive level of research in chromatin biology along with genome-wide tools to elucidate complexes, enzymatic function and chromatin occupancy, we may soon have enough information about real complexes and the behavior of their component readers and writers to simulate the chromatin computation that occurs in cells, and to learn some of the pieces of data that we are still missing.

The idealized chromatin model may serve as a starting point for a new way of building DNA-based computer using chromatin modifications for a read/write machine. A chromatin computer would operate on a fixed DNA sequence, and use histone and nucleotide modifications as the writable symbols. To engineer a chromatin computer based on this insight, the rules would be implemented in designer chromatin modification complexes built from naturally-occurring parts (protein domains). In an early proof of concept, researchers used human polycomb chromatin protein and homologs from other species to construct modular synthetic transcription factors that recognize H3K27me3 and switch silenced genes on [53]. This designed complex re-expressed tumor suppressor p16 (CDKN2A) and other loci in U2OS osteosarcoma cells.

The semantics and the value of the histone code concept have been the subject of debate, in particular the question of whether histone modifications carry much useful information beyond what can be inferred from transcription factor logic [54], [55], [56]. Here I show a remarkably straightforward mapping to a model of computation. It may be that biological chromatin modifications carry out actions (or passively reflect other processes) that are simple enough to be described as a direct mapping from observable patterns of marks to some functional readout. Yet it is tempting to hypothesize that considering chromatin modifications to be intermediate memory state in an ongoing computation will yield important biological insights.

## Methods

### Overview of proof that the chromatin computer is Turing complete

Here I prove that a chromatin computer can compute any computable function. I do this by defining a reversible mapping from any Turing machine to a chromatin computer, and from each Turing configuration (called an Instantaneous Description by Hopcroft and Ullman [5]) to a corresponding chromatin configuration. We then show that running the chromatin computer results in a chromatin configuration that maps back to the Turing configuration that would have been achieved by running the Turing machine, and that the chromatin computer halts whenever the Turing machine halts. The trick to the mapping is to transform each Turing tape cell to a nucleosome, with extra nucleosome positions to store the current state and the current location of the read/write head. Moving left or right along the Turing tape is accomplished on the chromatin by moving these state and head-location marks to adjacent nucleosomes.

### Definition of a Turing Machine

A Turing machine is defined by the 7-tuple 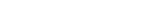, with the following elements:

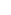
, a finite, non-empty set of states

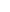
, a finite, non-empty set of tape symbols

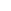
 is the blank symbol, and the only symbol allowed to be represented infinitely many times on the tape. It is an element of 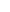

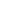
, the set of input symbols (a subset of 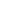)

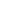
, the start state (an element of 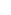)

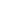
, the set of final or accepting states (a subset of 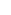)

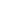
, a transition function 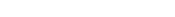. A deterministic Turing machine has at most one rule that is applicable for any given state and tape symbol.

A Turing machine operates on an infinitely long tape. Each location (cell) on the tape contains a symbol. A configuration of the Turing machine captures all the information that changes as the Turing machine executes its program: the configuration includes symbols written in each cell on the tape, the position of the read/write head, and the current state. We will write a Turing machine configuration as 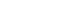, where 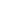 is an infinite vector of symbols written on the tape, 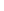 is an integer representing the location of the read/write head, and 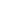 is the current state.

In the initial configuration, a finite number of cells on the tape can be written with symbols from 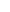; the remaining cells are blank. A read/write head is located at a given tape position at the start of computation. The machine has starting state 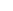.

At each step in the computation, the matching rule from 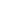 is applied to determine the new configuration from the current configuration. For example, if the head sees symbol 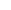 and is in state 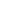, then it would find a rule whose left hand side specified that state and symbol, such as 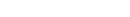. This rule says to change to state 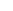, write the symbol 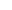 at the current location, and move the read/write head one cell to the left. If the new state is a final state, then computation halts.

### Mapping from any Turing configuration to a chromatin computer configuration

To prove that a chromatin computer can compute anything computable by a deterministic Turing machine, we will show that any Turing machine can be mapped to a chromatin computer, and that the computation performed by the chromatin computer results in a final configuration that can be uniquely mapped back to the final configuration that would be achieved by the Turing machine.

First we map a Turing configuration (tape symbols, head location and state) to 3-chromatin. Each cell of the Turing tape is mapped to one nucleosome. The three positions of each nucleosome will be used as follows:

1. Position 1 indicates the position of the read/write head on the Turing tape and may take one of two values: *B* or *H*. Because the read/write head exists at exactly one position, one and only one position on the entire chromatin tape will be marked with *H*; the rest are blank.
2. Position 2 is blank except when the Turing tape head is at the corresponding position on the Turing tape. In that case, Position 2 contains a mark representing the Turing machine state.
3. Position 3 contains a mark representing the Turing tape symbol written at that location.

This mapping is reversible: the single nucleosome marked with *H* maps to the read/write head location; the second position at that nucleosome maps back to the corresponding state, and the marks at the third positions all map back to the original symbols on the Turing tape.

A “**Turing-mappable**” configuration of the CC can be mapped back to a Turing configuration and meets these conditions:

1. The first position of every nucleosome is marked with *B*, except for the first position of some nucleosome *i*, which is marked with *H*.
2. The second position of every nucleosome is marked with *B*, except that the second position of nucleosome *i* is marked with an element *q*of *Q_CC_*
3. The third position of every nucleosome is marked with an element of 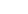.

We write a Turing-mappable CC configuration as 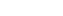, where 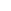 is the vector of marks in the third position, *i* is an integer representing the chromatin location of the nucleosome marked with *H*, and *q* is the mark at the second position of nucleosome *i*. A Turing-mappable CC configuration maps in a one-to-one mapping to the Turing configuration 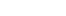. The mapping is one-to-one because the 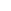 vectors are isomorphic, the position *i* is identical, and *q* is isomorphic.

Note that while CC's in general are non-deterministic, a deterministic Turing machine maps to a deterministic CC: if there is only one applicable Turing rule for a given configuration, then that translates to exactly one applicable rule in the CC.

### Construction of a CC that implements any Turing machine

The chromatin computer implementing the Turing machine is specified as follows:

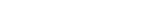
. 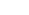 is a set of marks having a one-to-one mapping to the Turing machine states, augmented by 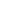, the blank mark. 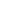 is a set of marks having a one-to-one mapping to the Turing machine tape symbols. 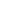 is the mark representing the location of the read/write head.

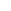
 represents absence of any chromatin mark; it is used to represent the blank symbol on the Turing tape as well as the absence of the read/write head in Position 1 or the absence of a state specification at Position 2. 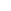 is an element of 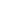 and of 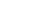, and therefore of 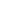.
3. The transition function 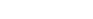, is constructed from the Turing machine's transition function. 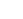 is the 9-dimensional space where each dimension is the set 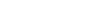. Actually, in our mapping from Turing configuration to CC configuration, the domain of *R* is 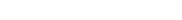, where the * indicates the option of using a wildcard, as before. The symbol that the Turing rule declares should be written is translated to a mark at the third position of the middle nucleosome. The read/write head movement is translated to moving the *H* mark to the adjacent nucleosome. The change of state becomes erasing the state mark from the middle nucleosome and writing the new state mark at the adjacent nucleosome. In other words,
  - Each “move left” Turing machine rule 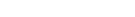 is mapped to the CC rule
BB* Hq_1_x_1_ BB* 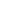 Hq_2_- BBx_2_ ---
  - Each “move right” Turing machine rule 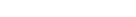 is mapped to the CC rule
BB* Hq_1_x_1_ BB* 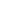 --- BBx_2_ Hq_2_-

### Proof that the chromatin computer implements the Turing machine

We show by induction that at each step of the CC computation, the configuration of the chromatin tape is Turing-mappable and is isomorphic to the state of the Turing tape after the same number of Turing machine steps, and that the CC will halt if and when the Turing machine halts.

The base case is the isomorphism between the initial configurations of the machines. 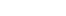 maps to 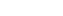, and as shown above, this mapping is isomorphic (reversible).

For the induction, we assume that after 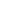 steps, the chromatin configuration is isomorphic to the Turing configuration after 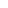 steps. Now we need to show that after the 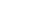 ^th^ step, the configurations remain isomorphic. Assume that the Turing rule that applies at this step is 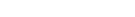. The corresponding CC rule would be BB* Hq_1_x_1_ BB* 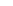 Hq_2_- BBx_2_ ---. To show isomorphism, we need to show that the tape symbols, read/write head position and state map 1-to-1 to the corresponding chromatin marks.

The only symbol to change on the Turing tape is the symbol in the cell at the read/write head (position 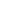 in the chromatin tape), which is changed from 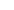 to 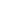. In the chromatin, the only 3^rd^-position mark to change is the one having the *i* ^th^ nucleosome marked with *H* at the first position. This changes from 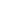 to 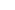. Because the vector of third-position marks changes in the same way and at the same position as the Turing tape, these configuration elements are isomorphic.

The read/write head position moves from *i* to 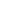, as specified by the *L* in the Turing rule. By the corresponding CC rule, the *H* in the chromatin at nucleosome *i* is changed to *B*, and the *B* at nucleosome 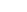 is changed to *H*. Thus the configuration element corresponding to the read/write head position is isomorphic.

The Turing machine state changes from 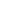 to 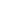. The mark 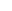 at position 2 of chromosome *i* is changed to a blank, and the blank at position 2 of chromosome 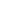 is changed to 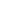. Thus by applying the CC rule to the starting configuration 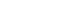 we end up with the Turing-mappable CC configuration 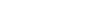, which maps back to the TM configuration 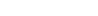, exactly the configuration obtained by applying the TM rule to the starting TM configuration. Thus we have shown that the chromatin and Turing configurations are isomorphic at step 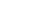, and by induction to all steps of the computation.

Finally, we show that the CC halts when the TM halts. The TM halts when a TM rule is applicable that moves the TM to a state in 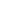. After applying this rule, there is no further TM rule in 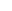 that can apply because 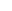 is defined to exclude any rule from matching a state in the final set 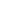. Therefore, no analogous rule in 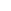 would have a mark mapping from that final state on the left hand side, and so no CC rule would apply, and the CC would also halt.

## Supporting Information

Text S1 **Additional notes; examples of biological read/write complexes; a lower bound on the size of the human chromatin computer; chromatin computer solution to the Hamiltonian Path Problem; perl script to simulate chromatin computer.**
(PDF)
