# Supplementary material for: Chromatin Computation
Source: PLoS One. 2012 May 2;7(5):e35703. doi: 10.1371/journal.pone.0035703 (PMC3342293; doi:10.1371/journal.pone.0035703)
Supplement: Text S1 — Additional notes; examples of biological read/write complexes; a lower bound on the size of the human chromatin computer; chromatin computer solution to the Hamiltonian Path Problem; perl script to simulate chromatin computer. (PDF) [file pone.0035703.s001.pdf]

Text S1. Supporting Text

Contents

Additional notes on chromatin computers 1
Additional example of chromatin computer operation 1
Note on nondeterminism 2
Examples of biological read/write complexes 3
A lower bound on the size of the human chromatin computer 5
Chromatin computer solution to Hamiltonian Path Problem 6
Input chromatin tape 6
Rules 6
Simulation: finding a Hamiltonian path 9
Simulation: flagging a path with repeated vertices 12
Repeated simulation until success 13
Additional rules to find the Hamiltonian path in a single run 14
Perl script to simulate chromatin computer 15
run_cc.pl 15
rules.pl 20
chromatin.pl 23
References 28

Additional notes on chromatin computers

Additional example of chromatin computer operation

The (**7**,**3**,**3**)-CC rule

`BB* NQP BB* → QR- BBS --T`

describes the reading and writing of 9 chromatin positions, arranged as the **3** positions in each of **3** adjacent nucleosomes. The **7** possible marks are *M* = {B, N, Q, P, R, S, T}. By convention, we included spaces in the read and write specifications to indicate the different nucleosomes. The rule only applies if the left-hand side matches the marks at 3 adjacent nucleosomes. In this rule, 7 of the 9 positions are specified, and two are wildcards, allowing any mark. The rule states that in the first nucleosome, the mark at the 3rd position should not be changed, and the blank positions 1 and 2 should be written with marks Q and R. In the second nucleosome, the marks in the first two positions, N and Q, should be

removed (overwritten with the “blank” character B), and the P in the third position should be changed to an S. In the third nucleosome, the blanks in positions 1 and 2 should remain, and the third position should be changed to a T. The left hand side of this rule can match $|M|^2$ different 9-mers (sets of 3 adjacent nucleosomes) because of the two wild card symbols. The right hand side of this rule could also be written `QR- BBS BBT`.

Note on nondeterminism

A chromatin computer is in general nondeterministic because a rule may match at multiple locations, and because more than one rule could match at a given location. A deterministic chromatin computer has at most one rule that matches anywhere along the chromatin at each time step. The proof of Turing completeness constructs a reversible mapping from a deterministic Turing machine to a chromatin computer, which is therefore also deterministic.

Examples of biological read/write complexes

To show that real biological chromatin-modifying complexes are capable of carrying out interesting computations, we can ask whether there are many that have multiple read/write functions. While this is a field of active research, there are indeed many known examples. In the Table below, I extract 39 examples from PINdb, a database of nuclear protein complexes [1]. For each, I list the PINdb name for the complex, along with the protein components that are erasers (which can be thought of as writers of a "blank" mark), writers or readers. Many of the proteins have multiple read/write domains, which would increase the valency of the components in which it operates. This table illustrates the point that a given protein may participate in several different complexes; it is likely that the combinatorics of protein inclusion in these complexes means that while I've listed 39 complexes here, there could be hundreds of complexes with at least two read or write components, and possibly far more. Not listed here are the scaffolding or connector proteins that hook the effector proteins together; there are many of these as well.

This list of protein complexes is an underestimate in several ways. For example, there are vastly many more complexes that contain transcription factors. There are hundreds of DNA-binding transcription factors [2]. Each one carries out its function in association with other proteins – in other words, it plays the reader role in one or more effector complexes.

Complex	Erasers (HDMs, HDACs)	Writers (HMTs, DNMTs, HATs)	Readers (bromodomains, PHD, PWWP, chromodomains, MBD)
NUMAC		CARM1	SMARCA4
GST-Smad2		CREBBP, NCOA3	CREBBP, SMARCA4, TRIM33
DNMT3B	HDAC1	DNMT3B	DNMT3B
AF9.com	HDAC1, HDAC2	DOT1L, TAF1, TAF5	CBX8, MLLT10, TAF1, TAF3
E2F6.com-1		EHMT1, EHMT2	CBX3
CtBP	HDAC1, HDAC2, KDM1A	EHMT1, EHMT2	CBX4, CDYL
SIRT1-LSD1	HDAC1, HDAC2, KDM1A, SIRT1	EHMT1, EHMT2	
Suv39h1		EHMT1, EHMT2, SETDB1	CBX1, CBX3, CBX5, SETDB1, TRIM28
TIP60		EPC1, ING3, KAT5	BRD8, ING3
DMAP1		EPC1, ING3, SRCAP	BRD8, ING3
STAGA		KAT2A, SF3B3, SUPT3H, SUPT7L, TADA1, TADA3, TAF10, TAF12, TAF5L, TAF6L, TAF9, USP22	KAT2A

PCAF		KAT2B, SUPT3H, TADA2A, TADA3, TAF10, TAF12, TAF5L, TAF6L, TAF9	KAT2B
ING5-TAP		KAT6A, KAT6B, KAT7	BRD1, BRPF1, BRPF3, ING5, KAT6A, KAT6B, PHF15, PHF16, PHF17
ING4-TAP		KAT7	ING4, PHF15, PHF16, PHF17
NSL		KAT8	PHF20
MLL1-WDR5		KAT8, MLL, TAF1, TAF9	CHD8, MLL, PHF20, TAF1
ALR	KDM6A	MLL2	MLL2
PTIP.com	KDM6A	MLL2, MLL3	MLL2, MLL3
Fl-BAF45		PRMT5	BRD7, DPF2, PBRM1, SMARCA2, SMARCA4
Fl-BAF57		PRMT5	BRD7, DPF2, PBRM1, SMARCA2, SMARCA4
PRC1L4		PRMT5	CBX3
Fl-hBrm		PRMT5	SMARCA2
CFP1		SETD1A	CXXC1
Set1B		SETD1B	CXXC1
ING2-TAP	HDAC1, HDAC2	SF3B3	ING2
N-CoR-1		SF3B3, SRCAP	SMARCA4, TRIM28
DAB		TAF1, TAF10, TAF12, TAF5, TAF9	TAF1, TAF3
ICEN	HDAC1		CBX3, CBX8, PHIP, RSF1
BAHD1	HDAC1, HDAC2		CBX3, TRIM28
NRD	HDAC1, HDAC2, KDM1A		CHD3, CHD4
HDAC2	HDAC1, HDAC2, KDM1A, MTA2		CHD3, CHD4, PHF21A
NuRD.1	HDAC1, HDAC2		CHD4

LARC	HDAC1, HDAC2, MTA2		CHD4, DPF2, MBD2, MBD3, SMARCA4
HDAC1	HDAC1, KDM1A		CHD4, MBD2, MBD3
emerin C32	HDAC1, HDAC3		CHD4, TRIM28
Sin3-CI	HDAC1, HDAC2		ING1, SMARCA4
MTA1-HDAC	HDAC1, HDAC2		MBD3
BHC	HDAC1, HDAC2, KDM1A		PHF21A
LSD1.com	HDAC1, KDM1A		PHF21A, PHF21B
ELL.com	HDAC1, HDAC2, KDM1A, KDM6A, UTY		

Table S 1. Some chromatin-modifying complexes from PINdb

A lower bound on the size of the human chromatin computer

How much chromatin memory is there in a human cell?

There are approximately 3,000,000,000 base pairs in the human genome [3]. There are 147 nucleotides wrapped around one nucleosome [4], and the linker region is around 10 additional nucleotides [5]. Davey et al give the average length between nucleosomes as 157-240 base pairs [6]. With some of the genome presumably nucleosome-free, I'll take 300 base pairs as a reasonable upper estimate for the length of DNA covered by a nucleosome, on average.

In Figure 2 of their review article, Zamudio and colleagues list 32 histone modification sites across H2A, H2B, H3 and H4 [7]. Each nucleosome contains two copies of each histone; therefore, there are 64 modification sites. I will assume that each position can have only one modification, although we know that some positions can be marked with one of several marks (such as one methyl group, two methyl groups, three methyl groups, acetylation, etc.).

Putting these numbers together, I arrive at 80 megabytes as a lower estimate of the amount of writable memory in human chromatin.

Size	Units	Item
3,000,000,000	base pairs	Size of genome
300	base pairs	length of region covered by a nucleosome, allowing for some nucleosome-free regions
10,000,000	nucleosomes	Therefore, number of nucleosomes in genome
64	locations	Number of modifiable locations on each nucleosome

2	marks	Number of possible marks at each position (marked or not marked)
2^{64}	mark combinations	Number of possible different values (mark combinations) taken by one nucleosome.
64	bits	Number of bits per nucleosome
8	bytes	Number of bytes per nucleosome
80,000,000	bytes	Number of bytes per genome
152,000	bytes	Amount of onboard memory in the Apollo mission that got astronauts to the moon (www.doneyles.com/LM/Tales.html)

Table S 2. Back-of-the-envelope numbers for length of biological chromatin

Chromatin computer solution to Hamiltonian Path Problem

Just as Adleman generated many pieces of DNA, each representing a path through the graph, we start with many pieces of chromatin, each of which will represent a path through the graph. (We could also put all these lengths on a single stretch of chromatin, and separate them with insulator nucleosomes with marks that do not ever change.) Each chromatin tape has seven nucleosomes, each with six positions.

Input chromatin tape

The initial configuration of each piece of chromatin is as follows:

```
000000 BBBBBB BBBBBB BBBBBB BBBBBB BBBBBB BBBBBB
```

Each nucleosome represents one vertex in the path. The first position at each nucleosome indicates which vertex is at that point in the path; it is either blank or a digit from 0 to 6. The remaining 5 positions will be used to check whether vertices 1 through 5 are visited exactly once, and take one of the values {B, 0, 1, F}. A "0" indicates that the corresponding vertex has not been seen in the path so far; a "1" indicates that it has been seen once; and an "F" indicates that it has been seen more than once. The 7th nucleosome of a valid path should have the configuration 611111, because the path should end at vertex 6 and each of the vertices 1 through 5 should have be seen exactly once.

Rules

Fourteen rules implement edge traversal from one vertex to another. For example, the following rule allows traversal of the edge from vertex 2 to vertex 3:

```
2***** B***** → ------ 3-----
```

Applying these rules to the 7-nucleosome initial configuration will result in a random path of up to length 7 through the graph. Some paths will be shorter than 7, and these we will not consider further as they will not lead to a solution. These path-constructing rules are analogous to the ligation in Adleman's solution.

Adleman solved the problem of checking that every node had been visited by doing affinity purification to check for the presence of each vertex. We can perform this check directly in the CC program. The following rules check that we have one and only one instance of vertex 1 in the path, and propagate the

necessary information from left to right along the nucleosomes representing the path. The “F” (for “Fail”) in the one rule below indicates that too many ones have been visited.

```
*0**** 1B**** → ------ -1----
*0**** 2B**** → ------ -0----
*0**** 3B**** → ------ -0----
*0**** 4B**** → ------ -0----
*0**** 5B**** → ------ -0----
*0**** 6B**** → ------ -0----
*1**** 1B**** → ------ -F----
*1**** 2B**** → ------ -1----
*1**** 3B**** → ------ -1----
*1**** 4B**** → ------ -1----
*1**** 5B**** → ------ -1----
*1**** 6B**** → ------ -1----
```

Similar rules check for a single instance of each of the other vertices, using nucleosome positions 3 through 6. The complete rule set is given below.

A nucleosome with the marks `611111` indicates the existence of a correct path. The following rule indicates success:

```
****** 611111 → ------ 6SSSSS
```

To read out the path after computation, read the nucleosome tape, looking at the first positions of each nucleosome. As described in the paper, we can augment the definition of the chromatin computer to allow output signaling (like gene expression) to indicate that the computation can halt because the current path is a valid Hamiltonian path, or else it has been found to be invalid. This can be used to bring computation to a halt. In the simulator, we assume that any rule with an “S” or an “F” in the right hand side brings computation to a halt. The simulator also halts, of course, if there is no applicable rule that can operate anywhere on the chromatin.

If a chromatin tape has an invalid path visiting a path more than once, then it will contain a nucleosome with the F symbol. Computation halts if no more rules apply (this happens when vertex 6 is reached because there are no further edges that can be traversed), or if a halt rule is encountered.

Note that an alternate way to solve this problem would be to add rules that erase the tape back to the first nucleosome if an invalid path is found, to allow reuse of the same chromatin tape. However, the solution presented here is simpler (in terms of the number of rules), and takes advantage of parallelism if it were available.

```
## Edge traversal
0***** B***** --> ------ 1-----
0***** B***** --> ------ 3-----
0***** B***** --> ------ 6-----
1***** B***** --> ------ 2-----
1***** B***** --> ------ 3-----
2***** B***** --> ------ 1-----
```

```
2***** B***** --> ------ 3-----
3***** B***** --> ------ 2-----
3***** B***** --> ------ 4-----
4***** B***** --> ------ 1-----
4***** B***** --> ------ 5-----
5***** B***** --> ------ 1-----
5***** B***** --> ------ 2-----
5***** B***** --> ------ 6-----

## Check for one and only one visit to vertex 1
*0**** 1B**** --> ------ -1----
*0**** 2B**** --> ------ -0----
*0**** 3B**** --> ------ -0----
*0**** 4B**** --> ------ -0----
*0**** 5B**** --> ------ -0----
*0**** 6B**** --> ------ -0----
*1**** 1B**** --> ------ -F----
*1**** 2B**** --> ------ -1----
*1**** 3B**** --> ------ -1----
*1**** 4B**** --> ------ -1----
*1**** 5B**** --> ------ -1----
*1**** 6B**** --> ------ -1----

## Check for one and only one visit to vertex 2
**0*** 2*B*** --> ------ --1---
**0*** 1*B*** --> ------ --0---
**0*** 3*B*** --> ------ --0---
**0*** 4*B*** --> ------ --0---
**0*** 5*B*** --> ------ --0---
**0*** 6*B*** --> ------ --0---
**1*** 2*B*** --> ------ --F---
**1*** 1*B*** --> ------ --1---
**1*** 3*B*** --> ------ --1---
**1*** 4*B*** --> ------ --1---
**1*** 5*B*** --> ------ --1---
**1*** 6*B*** --> ------ --1---

## Check for one and only one visit to vertex 3
***0** 3**B** --> ------ ---1--
***0** 1**B** --> ------ ---0--
***0** 2**B** --> ------ ---0--
***0** 4**B** --> ------ ---0--
***0** 5**B** --> ------ ---0--
***0** 6**B** --> ------ ---0--
***1** 3**B** --> ------ ---F--
***1** 1**B** --> ------ ---1--
***1** 2**B** --> ------ ---1--
***1** 4**B** --> ------ ---1--
***1** 5**B** --> ------ ---1--
***1** 6**B** --> ------ ---1--

## Check for one and only one visit to vertex 4
```

```
****0* 4***B* --> ------ ----1-
****0* 1***B* --> ------ ----0-
****0* 2***B* --> ------ ----0-
****0* 3***B* --> ------ ----0-
****0* 5***B* --> ------ ----0-
****0* 6***B* --> ------ ----0-
****1* 4***B* --> ------ ----F-
****1* 1***B* --> ------ ----1-
****1* 2***B* --> ------ ----1-
****1* 3***B* --> ------ ----1-
****1* 5***B* --> ------ ----1-
****1* 6***B* --> ------ ----1-

## Check for one and only one visit to vertex 5
*****0 5****B --> ------ -----1
*****0 1****B --> ------ -----0
*****0 2****B --> ------ -----0
*****0 3****B --> ------ -----0
*****0 4****B --> ------ -----0
*****0 6****B --> ------ -----0
*****1 5****B --> ------ -----F
*****1 1****B --> ------ -----1
*****1 2****B --> ------ -----1
*****1 3****B --> ------ -----1
*****1 4****B --> ------ -----1
*****1 6****B --> ------ -----1

## Success
****** 611111 --> ------ 6SSSSS
```

Simulation: finding a Hamiltonian path

Here I give an example of a successful sequence of application of the rules to find a Hamiltonian path. This is output from the perl script in "verbose" mode. (The actual run of the simulator used an input tape that had the insulator nucleosome `IIIIII` on the right hand side of the nucleosomes displayed below, which I've removed for readability.) The first line shows the initial configuration of the chromatin tape. The second line shows the "read" specification or left hand side of the first rule to be applied, and the third line shows the "write" specification or right hand side. The rule looks for a 0 in the first position of a nucleosome, and a B in the first position of the next nucleosome. It then writes a 1 at the first position of the second nucleosome. This implements the traversal of the edge from vertex 0 to vertex 1.

```
000000 BBBBBB BBBBBB BBBBBB BBBBBB BBBBBB BBBBBB
0***** B*****
------ 1-----
000000 1BBBBB BBBBBB BBBBBB BBBBBB BBBBBB BBBBBB
       1***** B*****
       ------ 2-----
000000 1BBBBB 2BBBBB BBBBBB BBBBBB BBBBBB BBBBBB
              2***** B*****
              ------ 3-----
000000 1BBBBB 2BBBBB 3BBBBB BBBBBB BBBBBB BBBBBB
```

The next rule writes the number of times vertex 1 has been visited, when we are at the second vertex in the path. (The answer is 1.) The rule after that records the fact that vertex 5 has been visited 0 times at that point in the path. We then continue on, with a matching rule randomly selected, building the path and checking for repeated vertices.

```
000000 1BBBBB 2BBBBB 3BBBBB BBBBBB BBBBBB BBBBBB
*0**** 1B****
------ -1----
000000 11BBBB 2BBBBB 3BBBBB BBBBBB BBBBBB BBBBBB
*****0 1****B
------ -----0
000000 11BBB0 2BBBBB 3BBBBB BBBBBB BBBBBB BBBBBB
**0*** 1*B***
------ --0---
000000 110BB0 2BBBBB 3BBBBB BBBBBB BBBBBB BBBBBB
****0* 1***B*
------ ----0-
000000 110B00 2BBBBB 3BBBBB BBBBBB BBBBBB BBBBBB
                     3***** B*****
                     ------ 4-----
000000 110B00 2BBBBB 3BBBBB 4BBBBB BBBBBB BBBBBB
***0** 1**B**
------ ---0--
000000 110000 2BBBBB 3BBBBB 4BBBBB BBBBBB BBBBBB
       *****0 2****B
       ------ -----0
000000 110000 2BBBB0 3BBBBB 4BBBBB BBBBBB BBBBBB
       **0*** 2*B***
       ------ --1---
000000 110000 2B1BB0 3BBBBB 4BBBBB BBBBBB BBBBBB
                            4***** B*****
                            ------ 5-----
000000 110000 2B1BB0 3BBBBB 4BBBBB 5BBBBB BBBBBB
       ****0* 2***B*
       ------ ----0-
000000 110000 2B1B00 3BBBBB 4BBBBB 5BBBBB BBBBBB
       ***0** 2**B**
       ------ ---0--
000000 110000 2B1000 3BBBBB 4BBBBB 5BBBBB BBBBBB
              **1*** 3*B***
              ------ --1---
000000 110000 2B1000 3B1BBB 4BBBBB 5BBBBB BBBBBB
              ***0** 3**B**
              ------ ---1--
000000 110000 2B1000 3B11BB 4BBBBB 5BBBBB BBBBBB
              ****0* 3***B*
              ------ ----0-
000000 110000 2B1000 3B110B 4BBBBB 5BBBBB BBBBBB
                                   5***** B*****
                                   ------ 6-----
000000 110000 2B1000 3B110B 4BBBBB 5BBBBB 6BBBBB
                     ***1** 4**B**
                     ------ ---1--
000000 110000 2B1000 3B110B 4BB1BB 5BBBBB 6BBBBB
                     ****0* 4***B*
```

```
                     ------ ----1-
000000 110000 2B1000 3B110B 4BB11B 5BBBBB 6BBBBB
       *1**** 2B****
       ------ -1----
000000 110000 211000 3B110B 4BB11B 5BBBBB 6BBBBB
              *****0 3****B
              ------ -----0
000000 110000 211000 3B1100 4BB11B 5BBBBB 6BBBBB
              *1**** 3B****
              ------ -1----
000000 110000 211000 311100 4BB11B 5BBBBB 6BBBBB
                     **1*** 4*B***
                     ------ --1---
000000 110000 211000 311100 4B111B 5BBBBB 6BBBBB
                     *****0 4****B
                     ------ -----0
000000 110000 211000 311100 4B1110 5BBBBB 6BBBBB
                            **1*** 5*B***
                            ------ --1---
000000 110000 211000 311100 4B1110 5B1BBB 6BBBBB
                     *1**** 4B****
                     ------ -1----
000000 110000 211000 311100 411110 5B1BBB 6BBBBB
                            ****1* 5***B*
                            ------ ----1-
000000 110000 211000 311100 411110 5B1B1B 6BBBBB
                            *****0 5****B
                            ------ -----1
000000 110000 211000 311100 411110 5B1B11 6BBBBB
                            ***1** 5**B**
                            ------ ---1--
000000 110000 211000 311100 411110 5B1111 6BBBBB
                                   ****1* 6***B*
                                   ------ ----1-
000000 110000 211000 311100 411110 5B1111 6BBB1B
                                   **1*** 6*B***
                                   ------ --1---
000000 110000 211000 311100 411110 5B1111 6B1B1B
                                   *****1 6****B
                                   ------ -----1
000000 110000 211000 311100 411110 5B1111 6B1B11
                                   ***1** 6**B**
                                   ------ ---1--
000000 110000 211000 311100 411110 5B1111 6B1111
                            *1**** 5B****
                            ------ -1----
000000 110000 211000 311100 411110 511111 6B1111
                                   *1**** 6B****
                                   ------ -1----
000000 110000 211000 311100 411110 511111 611111
                                   ****** 611111
                                   ------ 6SSSSS
000000 110000 211000 311100 411110 511111 6SSSSS
```

The final rule applied writes the “S” symbols that trigger a halt in the simulation.

Simulation: flagging a path with repeated vertices

In this simulation, the sequence of applied rules leads to a failure state

```
000000 BBBBBB BBBBBB BBBBBB BBBBBB BBBBBB BBBBBB
0***** B*****
------ 3-----
000000 3BBBBB BBBBBB BBBBBB BBBBBB BBBBBB BBBBBB
****0* 3***B*
------ ----0-
000000 3BBB0B BBBBBB BBBBBB BBBBBB BBBBBB BBBBBB
**0*** 3*B***
------ --0---
000000 3B0B0B BBBBBB BBBBBB BBBBBB BBBBBB BBBBBB
*0**** 3B****
------ -0----
000000 300B0B BBBBBB BBBBBB BBBBBB BBBBBB BBBBBB
       3***** B*****
       ------ 4-----
000000 300B0B 4BBBBB BBBBBB BBBBBB BBBBBB BBBBBB
       **0*** 4*B***
       ------ --0---
000000 300B0B 4B0BBB BBBBBB BBBBBB BBBBBB BBBBBB
       *0**** 4B****
       ------ -0----
000000 300B0B 400BBB BBBBBB BBBBBB BBBBBB BBBBBB
*****0 3****B
------ -----0
000000 300B00 400BBB BBBBBB BBBBBB BBBBBB BBBBBB
       *****0 4****B
       ------ -----0
000000 300B00 400BB0 BBBBBB BBBBBB BBBBBB BBBBBB
***0** 3**B**
------ ---1--
000000 300100 400BB0 BBBBBB BBBBBB BBBBBB BBBBBB
              4***** B*****
              ------ 1-----
000000 300100 400BB0 1BBBBB BBBBBB BBBBBB BBBBBB
              **0*** 1*B***
              ------ --0---
000000 300100 400BB0 1B0BBB BBBBBB BBBBBB BBBBBB
              *****0 1****B
              ------ -----0
000000 300100 400BB0 1B0BB0 BBBBBB BBBBBB BBBBBB
              *0**** 1B****
              ------ -1----
000000 300100 400BB0 110BB0 BBBBBB BBBBBB BBBBBB
                     1***** B*****
                     ------ 3-----
000000 300100 400BB0 110BB0 3BBBBB BBBBBB BBBBBB
                            3***** B*****
                            ------ 2-----
000000 300100 400BB0 110BB0 3BBBBB 2BBBBB BBBBBB
                     *1**** 3B****
                     ------ -1----
000000 300100 400BB0 110BB0 31BBBB 2BBBBB BBBBBB
       ***1** 4**B**
```

```
       ------ ---1--
000000 300100 4001B0 110BB0 31BBBB 2BBBBB BBBBBB
                            *1**** 2B****
                            ------ -1----
000000 300100 4001B0 110BB0 31BBBB 21BBBB BBBBBB
                                   2***** B*****
                                   ------ 3-----
000000 300100 4001B0 110BB0 31BBBB 21BBBB 3BBBBB
                                   *1**** 3B****
                                   ------ -1----
000000 300100 4001B0 110BB0 31BBBB 21BBBB 31BBBB
                     *****0 3****B
                     ------ -----0
000000 300100 4001B0 110BB0 31BBB0 21BBBB 31BBBB
              ***1** 1**B**
              ------ ---1--
000000 300100 4001B0 1101B0 31BBB0 21BBBB 31BBBB
                     ***1** 3**B**
                     ------ ---F--
000000 300100 4001B0 1101B0 31BFB0 21BBBB 31BBBB
```

Repeated simulation until success

We run the simulator many times, until we achieve success.   Each row belowshows the chromatin tape at the time that the computer halted due to either achieving a success state, a fail state (repeated vertices on the path), or no more rules matched (got to vertex 6 in the graph).

```
000000 30010B 201BBB 111BBB 2BFBBB 1BBBBB 2BBBBB
000000 600000 BBBBBB BBBBBB BBBBBB BBBBBB BBBBBB
000000 300100 4B0110 1B011B 2B11BB 3BBFBB 2BBBBB
000000 110000 31010B 41011B 51011B 1FB1BB 2BB1BB
000000 110000 310100 211100 311F00 411B10 511B11
000000 600000 BBBBBB BBBBBB BBBBBB BBBBBB BBBBBB
000000 110000 310B0B 410B1B 1FBBBB 3BBBBB 2BBBBB
000000 1100B0 21B0BB 1FBBBB BBBBBB BBBBBB BBBBBB
000000 110000 310100 2B1100 1B1100 2BF100 1BB1BB
000000 600000 BBBBBB BBBBBB BBBBBB BBBBBB BBBBBB
000000 300100 4B011B 1B011B 2B11BB 1B1BBB 2BFBBB
000000 300100 400110 500111 11B1BB 21BBBB 1FBBBB
000000 3001BB 20B1BB 3BBFBB BBBBBB BBBBBB BBBBBB
000000 600000 BBBBBB BBBBBB BBBBBB BBBBBB BBBBBB
000000 110000 310100 4BB11B 1BB11B 3BBF1B BBBBBB
000000 1B0000 3BB100 2BB100 3BBF0B BBBBBB BBBBBB
000000 110000 211000 311100 411110 511111 21F111
000000 300100 400110 110110 310F10 2B1B10 3B1B1B
000000 300100 201100 111100 21FB00 3BBBB0 4BBBBB
000000 300100 400110 500111 110111 211111 311F11
000000 110000 211000 311B00 21BB00 1FBBB0 3BBBB0
000000 1100B0 2110B0 3111B0 21F1BB 3BBBBB BBBBBB
000000 300100 201100 301F0B 2BBB0B 3BBBBB BBBBBB
000000 600000 BBBBBB BBBBBB BBBBBB BBBBBB BBBBBB
000000 600000 BBBBBB BBBBBB BBBBBB BBBBBB BBBBBB
000000 1100B0 310BB0 2B1BBB 3B1BBB 2BFBBB 1BBBBB
```

```
000000 300100 400110 110110 211110 1B1110 3BBFB0
000000 300100 400110 1B0110 3B0FB0 2B1BBB 3BBBBB
000000 11B000 31BB00 41BBB0 1FBBBB 3BBBBB 2BBBBB
000000 600000 BBBBBB BBBBBB BBBBBB BBBBBB BBBBBB
000000 300100 201100 1B1100 2BFB0B 1BBBBB 3BBBBB
000000 600000 BBBBBB BBBBBB BBBBBB BBBBBB BBBBBB
000000 600000 BBBBBB BBBBBB BBBBBB BBBBBB BBBBBB
000000 110000 31010B 2111BB 1F11BB 3BBBBB BBBBBB
000000 300100 2011B0 301FBB 4BBBBB 5BBBBB 2BBBBB
000000 600000 BBBBBB BBBBBB BBBBBB BBBBBB BBBBBB
000000 300100 400110 110110 21B110 31BF10 2BBBB0
000000 110000 310100 21110B 1FBB0B 3BBB0B 4BBBBB
000000 110000 2110BB 1F10BB 3B1BBB 4BBBBB 5BBBBB
000000 300100 4BB110 1BB1B0 2BB1B0 1BB1BB 3BBFBB
000000 300100 400110 500111 201111 111111 311F11
000000 30010B 4001BB 5B01BB 2BB1BB 3BBFBB 2BBBBB
000000 300100 2011B0 1111B0 31BFB0 41BBB0 5BBBBB
000000 600000 BBBBBB BBBBBB BBBBBB BBBBBB BBBBBB
000000 110000 211000 311100 21FB00 3BBBB0 2BBBB0
000000 300B00 40BB10 11BB10 21BBBB 1FBBBB BBBBBB
000000 300100 2011B0 3BBFBB 4BBBBB BBBBBB BBBBBB
000000 600000 BBBBBB BBBBBB BBBBBB BBBBBB BBBBBB
000000 600000 BBBBBB BBBBBB BBBBBB BBBBBB BBBBBB
000000 110000 211000 311100 21B10B 3BBF0B 4BBBBB
000000 110000 211000 311100 411110 511111 6SSSSS
```

The last chromatin configuration is the one that achieves success, showing that the correct order for visiting the nodes is 0,1,2,3,4,5,6.

Additional rules to find the Hamiltonian path in a single run

It is possible to solve the Hamiltonian path problem with a single stretch of 8 nucleosomes by adding rules to reset the chromatin state if we explore a path that repeats a vertex or gets to the finish too early. The starting state for this rule set has an insulator sequence at the right edge.

```
000000 BBBBBB BBBBBB BBBBBB BBBBBB BBBBBB BBBBBB IIIIII
```

In these rules, we use “X” instead of “F” for the symbol indicating that a path has visited a vertex more than once. When this happens, we want to reset the chromatin to its initial state and start over. “G” is used as a byproduct of tracking toward the right to make sure we erase the area we are using for memory; “H” is used to walk back to the left, erasing everything back to the starting point. The edge traversal and vertex counting rules are the same as before, but with “F” replaced with “X” (so that the perl script does not interpret it as a halting state). In addition we have the following rules:

```
## If stuck at 6, erase
6***** BBBBBB --> X----- ------

## If hit wall, turn around
1***** IIIIII --> H----- ------
2***** IIIIII --> H----- ------
```

```
3***** IIIIII --> H----- ------
4***** IIIIII --> H----- ------
5***** IIIIII --> H----- ------

## Walk failure right to the I wall.
X***** 1***** --> G----- X-----
X***** 2***** --> G----- X-----
X***** 3***** --> G----- X-----
X***** 4***** --> G----- X-----
X***** 5***** --> G----- X-----
X***** 6***** --> G----- X-----

## Bounce back if hit right end
X***** IIIIII --> H----- ------
X***** BBBBBB --> H----- ------

## Walk left to 0, erasing as you go
G***** H***** --> H----- BBBBBB
X***** H***** --> H----- BBBBBB
1***** H***** --> H----- BBBBBB
2***** H***** --> H----- BBBBBB
3***** H***** --> H----- BBBBBB
4***** H***** --> H----- BBBBBB
5***** H***** --> H----- BBBBBB
6***** H***** --> H----- BBBBBB
0***** H***** --> 000000 BBBBBB
```

Perl script to simulate chromatin computer

run_cc.pl

```
#!C:\Perl\bin\perl.exe

## run_cc_3.pl
##
## to run:
##  run_cc_3.pl --rules rules_flip_flop.txt --input initial_flip_flop.txt
##  run_cc_3.pl --rules complete_rules_hamiltonian_path2.txt --input
initial_chromatin_ham_path2.txt

## Run a chromatin computer

use strict;
use Getopt::Long;
require "rules.pl";
require "chromatin.pl";
our $help_text;

## ============= GLOBAL VARIABLES  =============

## Arguments to script
our ($help, $rules_file, $input_file, $verbose, $iters, $show);

## keys 'rules', 'k', 'n'
## $CC->{'rules'} is a pointer to an array.
```

```
our $CC;

## Cache for all the matches of rules to chromatin.
## One match is picked at random at each step.
## keys are integers for chromatin tape location, and rule index.
our $MATCH;

## =================== Argument Processing ===================

## for more information on using GetOptions, see
## http://www.perl.com/pub/a/2007/07/12/options-and-configuration.html
GetOptions('help' => \$help,
         'iters=i' => \$iters,
         'rules=s' => \$rules_file,
         'input=s' => \$input_file,
         'verbose' => \$verbose,
         'show' => \$show
         );

if (!$rules_file | !$input_file) {
  $help=1;
}

if (!$iters) {$iters = 1;}

## if (!$INNER) {$INNER = $default_inner;}

if ($help) {
  print "Usage at command line:\n";
  print " run_cc_3.pl --rules <filename> --input <filename> > outfile.txt\n";
  print " Arguments:\n";
  print "   --help         Prints this message\n";
  print "   --rules        Rules file, space delimited\n";
  print "   --input        Starting chromatin configuration file, tab
delimited\n";
  print "   --iters        Maximum number of runs (default 1)\n";
  print "   --verbose      Verbose mode\n";
  print "   --show         Show chromatin at every step\n";
  print $help_text;
  exit 1;
}

## ================= Top level ====================

read_rules($rules_file);
if ($verbose) {
  summarize_rules();
}
my $chromatin = read_chromatin($input_file);

## my ($done, $chromatin) = run_cc($chromatin);
## print("Finished with readout $done\n");
## print_chromatin($chromatin);

run_cc_many_times($chromatin, $iters);

## ================= Subroutines =================
```

```
sub check_nucleosome_lengths {
  my $k = length($_[0]);
  foreach my $nucleosome (@_) {
    if (length($nucleosome) != $k) {
      die("Nucleosome |$nucleosome| has a different length than |$_[0]|");
    }
  }
  $k;
}

sub run_cc_many_times {
  my $starting_chromatin = shift;
  my $n = get_n();
  my $k = get_k();
  my $iterations = shift;
  my $done = 0;
  my $i = 1;
  while (($i <= $iterations) && !$done) {
    my ($halt_state, $chromatin) =
run_cc(copy_chromatin($starting_chromatin));
##    if ($verbose) {
    print "$halt_state\t";
    print_chromatin($chromatin);
    print "\n";
##    }
    if ($halt_state eq "S") { ## success
      $done = 1;
      print_chromatin($chromatin);
      print("\nFinished in $i iterations.\n");
    }
    $i++;
  }
}

sub run_cc {
  my $chromatin = shift;
  ## Set up match cache;
  initialize_match_cache($chromatin);
  ## print_match_cache();
##   if ($verbose) {print_rules(); die();}
  ## repeatedly pick a random rule and a random location.
  ## Do not be dumb and keep trying things that don't work, though
  my $halt_state_p = 0;
  while (!$halt_state_p) {
    my ($rule_i, $chromatin_location) = random_matching_rule_location();
    if ($rule_i == -1) {
      ## print "NO MORE RULES MATCH\n";
      return(-1, $chromatin);
    }
    my $rule = get_nth_rule($rule_i);
    if (!match($rule, $chromatin_location, $chromatin)) {
      die("run_cc: cached rule/location does not seem to match");
    }
    ## if ($verbose) {
    ## print_chromatin_location($chromatin_location);
    ## print_rule($rule);
    ## }
```

```
    ($halt_state_p, $chromatin) = apply_rule($rule, $chromatin_location,
$chromatin);
    if ($verbose || $show) {
      print_rule_two_lines($rule, $chromatin_location, $chromatin);
      print_chromatin($chromatin);
      print "\n";
    }
  }
  return($halt_state_p, $chromatin);
}

## this could be more efficient:  just remove the locations that have
## just been processed, and recompute the matches for any window that
## overlaps that location.
sub update_match_cache {
  my $chromatin = shift;
  initialize_match_cache($chromatin);
}

sub initialize_match_cache {
  my $chromatin = shift;
  $MATCH = {};
  my @rules = get_rules();
  foreach my $i (0..$#rules) {
    my $rule = $rules[$i];
    foreach my $location (get_all_locations($chromatin)) {
      if (match($rule, $location, $chromatin)) {
      $MATCH->{$i}{$location} = 1;
      }
    }
  }
}

sub print_match_cache {
  print("\nMATCH CACHE:\n");
  foreach my $rule_i (keys %{$MATCH}) {
    foreach my $location (keys %{$MATCH->{$rule_i}}) {
      print("----------- MATCH ----------\n");
      print_rule(get_nth_rule($rule_i));
      print_location($location);
    }
  }
  print "\n";
}

sub match {
  my $rule = shift;
  my $chromatin_location = shift;
  my $chromatin = shift;
  my @chromatin_nucs = get_n_nucleosomes($chromatin, $chromatin_location);
  my @lhs = lhs($rule);
  if ($#lhs != $#chromatin_nucs) {  ## check number of chromosomes
    print_rule($rule);
    die ($#lhs+1 . "nucleosomes in the rule but " . $#chromatin_nucs+1 . "
nucleosomes in the chromatin");
  }
  my ($r, $c);
```

```
  foreach my $i (0..$#lhs) {
    $r = $lhs[$i];
    $c = $chromatin_nucs[$i];
    ## if ($verbose) {
    ## print("match location $chromatin_location, position $i: $r == $c?\n");
    ## }
    if (!($c =~ /$r/)) {
      return 0
    }
  }
  1;
}

## A rule containing S or F on the rhs is a halting rule
sub is_halt_rule {
  my $rule = shift;
  ##
  my @rhs = rhs($rule);
  foreach my $triplet (@rhs) {
    ## print($triplet->[2], "\n");
    if ($triplet->[2] eq "F") {
      if ($verbose) {print("FAIL\n");}
      return "F";
    }
    if ($triplet->[2] eq "S") {
      if ($verbose) {print("SUCCESS\n");}
      return "S";
    }
  }
  return 0;
}

## =================== RULES & CHROMATIN ==============

sub apply_rule {
  my $rule = shift;
  my $chromatin_location = shift;
  my $chromatin = shift;
  ## if ($verbose) {
  ## print("In subroutine apply_rule at location $chromatin_location.\n");
  ## }
  my $done=0;
  my @rhs = rhs($rule);
  $done = is_halt_rule($rule);
  ## do the replacement
  foreach my $triplet (@rhs) {
    my $nuci = $triplet->[0];
    my $chari = $triplet->[1];
    my $newchar = $triplet->[2];
    write_mark($chromatin, $chromatin_location,
             $nuci, $chari, $newchar);
  }
  ## if the nucleosome is within n-1 nucleosomes of the end,
  ## and it is no longer blank, then we need to add a blank nucleosome
  ## to the right hand edge.
  ## This can be done more efficiently, but this is easy for now.
  $chromatin = pad_chromatin($chromatin);
```

```
  update_match_cache($chromatin);
  return($done, $chromatin);
}

sub trim_line {
  my $line = shift;
  chomp $line;
  $line =~ s/\s+$//;
  $line =~ s/^\s+//;
  $line;
}


sub process_lhs_nucleosomes {
  my @nuc_regexps = ();
  foreach my $nuc (@_) {
    $nuc =~ s/\*/\./g;
    push(@nuc_regexps, $nuc);
  }
  @nuc_regexps;
}

## we are going to use these in this way:
## substr($string, $i, 1, $new_char)
sub process_rhs_nucleosomes {
  my @replacements = ();
  my $nuci = 0;
  foreach my $nuc (@_) {
    my $i = 0;
    foreach my $char (split //, $nuc) {
      if ($char ne "-") {
      push(@replacements, [$nuci, $i, $char]);
      }
      $i++;
    }
    $nuci++;
  }
  @replacements;
}
```

rules.pl

```
our $CC;

sub pick_rule {
  my $rules = $CC->{'rules'};
  my @rules = @{$rules};
  my $i = rand_int_in_range(0, $#rules);
  $rules[$i];
}

sub lhs {
  my $rule = shift;
  @{$rule->[0]};
}
```

```
sub rhs {
  my $rule = shift;
  @{$rule->[1]};
}

sub lhs_string {
  my $rule = shift;
  $rule->[2];
}

sub rhs_string {
  my $rule = shift;
  $rule->[3];
}

sub print_rule {
  my $rule = shift;
  my @lhs = lhs($rule);
  my @rhs = rhs($rule);
  print "RULE:\n";
  print "   LHS nucleosomes:", join(", ", @lhs), "\n";
  print "   RHS nucleosomes:\n";
  foreach my $item (@rhs) {
    print "      Nucleosome ", $item->[0], "; position ", $item->[1], ";
replacement ", $item->[2], "\n";
  }

  ## code here ******88
}

sub print_rules {
  foreach my $rule (get_rules()) {
    print_rule($rule);
  };
}

sub summarize_rules {
  print "k = " . get_k() . "; n = " . get_n() . "; ";
  print n_rules() . " rules.\n";
}

sub n_rules {
  my @rules = get_rules();
  return $#rules + 1;
}

sub get_rules {
  return @{$CC->{'rules'}};
}

sub get_nth_rule {
  my $n = shift;
  my @rules = get_rules();
  $rules[$n];
}

sub read_rules {
```

```
  my $file = shift;
  my @rules = ();
  open(IN, $file) || die("Could not read $file");
  while (my $line = <IN>) {
    $line = trim_line($line);
    if ($line && !($line =~ /^#/)) {
      process_rule($line);
    }
  }
  close(IN);
}

sub process_rule {
  my $line = shift;
  if (!$line =~ /-->/) {
    die("Could not find --> in line $line");
  }
##  if ($verbose) {
##    print("process_rule:  $line\n");
##  }
  my ($lhs, $rhs) = split("-->", $line);
  $lhs = trim_line($lhs);
  $rhs = trim_line($rhs);
  my @lhs_nucleosomes = split(/\s/, $lhs);
  my @rhs_nucleosomes = split(/\s/, $rhs);
  ## check all nucleosomes are the same length.
  disallow_blank_lhs(@lhs_nucleosomes);
  my $k1 = check_nucleosome_lengths(@lhs_nucleosomes, @rhs_nucleosomes);
  if ($CC->{'k'} && ($CC->{'k'} != $k1)) {
    die ("k should be " . $CC->{'k'} . " but in this rule it is $k1: $line");
  }
  else {
    $CC->{'k'} = $k1;
  }
  if ($#lhs_nucleosomes != $#rhs_nucleosomes) {
    die ("LHS and RHS of rule should have same number of nucleosomes");
  }
  if ($CC->{'n'}) {
    if ($CC->{'n'} != 1 + $#lhs_nucleosomes) {
      die("All rules should have the same number of nucleosomes");
    }
  }
  else {
    $CC->{'n'} = 1 + $#lhs_nucleosomes;
  }
  @lhs_nucleosomes = process_lhs_nucleosomes(@lhs_nucleosomes);
  @rhs_nucleosomes = process_rhs_nucleosomes(@rhs_nucleosomes);
##  if ($verbose) {
##    print("LHS ", join(", ", @lhs_nucleosomes), "\n");
##    print("RHS ", join(", ", @rhs_nucleosomes), "\n");
##  }
  ## *** will this work, pushing onto a cast list? ***
  push(@{$CC->{'rules'}},
       [\@lhs_nucleosomes, \@rhs_nucleosomes, $lhs, $rhs]);
}

sub disallow_blank_lhs {
```

```
  foreach my $nucleosome (@_) {
    if (!(nucleosome =~ /B+/)) {
      return 1;
    }
  }
  die "Rule LHS must have at least one non-blank character";
}

sub get_k {
  return $CC->{'k'};
}

sub get_n {
  return $CC->{'n'};
}

1;
```

chromatin.pl

```
## ============== CHROMATIN =============

our $verbose;
our $MATCH;

## $chromatin is a hashref with keys
##   'max'
##   'min'
##   'tape'

## Needs to be replaced ***
sub pick_chromatin_location {
  my $chromatin = shift;
  my $n = get_n();
  if (!$n) {
    die("pick_chromatin_location: missing argument n");
  }
  my @locations = get_all_locations($chromatin);
  my $start = rand_int_in_range($locations[0], $locations[$#locations]);
  $start;
}

sub all_matching_rule_locations {
  my @rule_locations = ();
  foreach my $rule_index (keys %{$MATCH}) {
    foreach my $location (keys %{$MATCH->{$rule_index}}) {
      push(@rule_locations, [$rule_index, $location]);
    }
  }
  @rule_locations;
}

sub random_matching_rule_location {
  my @rule_locations = all_matching_rule_locations();
  if ($#rule_locations == -1) {
    return (-1, -1);
  }
```

```
  else {
    my $rl = $rule_locations[rand_int_in_range(0, $#rule_locations)];
    return @{$rl};
  }
}

sub rand_int_in_range {
  my $low = shift;
  my $high = shift;
  int(rand($high - $low + 1)) + $low;
}

sub print_location {
  my $location = shift;
  print "Location: $location\n";
}

sub get_all_locations {
  my $chromatin = shift;
  my $n = get_n();
  return ($chromatin->{'min'} .. ($chromatin->{'max'}-$n+1));
}

sub get_nucleosome {
  my $chromatin = shift;
  my $location = shift;
  return $chromatin->{'tape'}{$location};
}

sub get_nucleosomes {
  my $chromatin = shift;
  my $from = shift;
  my $to = shift;
  my @nucs = ();
  foreach my $k ($from .. $to) {
    push(@nucs, $chromatin->{'tape'}{$k});
  }
  @nucs;
}

## ** Um... **
sub print_chromatin_location {
  my $location = shift;
  print("Chromatin location: $location\n");
}

## updated
sub chromatin_length {
  my $chromatin = shift;
  return ($chromatin->{'max'} - $chromatin->{'min'} + 1);
}

## updated
sub copy_chromatin {
  my $chromatin = shift;
  my $new;
  $new->{'min'} = $chromatin->{'min'};
```

```
  $new->{'max'} = $chromatin->{'max'};
  foreach my $i ($new->{'min'}..$new->{'max'}) {
    $new->{'tape'}{$i} = $chromatin->{'tape'}{$i};
  }
  $new;
}

sub write_mark {
  my $chromatin = shift;
  my $location = shift;
  my $nuci = shift;
  my $marki = shift;
  my $newmark = shift;
  ## my $old_nucleosome = get_nucleosome($chromatin, $location+$nuci, 1);
  substr($chromatin->{'tape'}{$location+$nuci}, $marki, 1) =  $newmark;
}

sub pad_left {
  my $chromatin = shift;
  my $n_blanks = shift;
  $chromatin->{'min'} = $chromatin->{'min'} - $n_blanks;
  foreach my $i ($chromatin->{'min'}..($chromatin->{'min'} + $n_blanks -1)) {
    $chromatin->{'tape'}{$i} = blank_nucleosome();
  }
  $chromatin;
}

sub pad_right {
  my $chromatin = shift;
  my $n_blanks = shift;
  $chromatin->{'max'} = $chromatin->{'max'} + $n_blanks;
  foreach my $i (($chromatin->{'max'}-$n_blanks+1)..$chromatin->{'max'}) {
    $chromatin->{'tape'}{$i} = blank_nucleosome();
  }
  $chromatin;
}

sub is_blank {
  my $string = shift;
  $string eq blank_nucleosome();
}

sub pad_chromatin {
  my $chromatin = shift;
  my $n = get_n();
  ## ----------- left end ------------
  my $left_i = $chromatin->{'min'};
  my $n_left_blanks = 0;
  CHECK_LEFT: foreach my $nuci ($left_i .. ($left_i + $n - 2)) {
    if (is_blank(get_nucleosome($chromatin, $nuci))) {
      $n_left_blanks++;
    }
    else {
      last CHECK_LEFT;
    }
  }
  if ($n_left_blanks < ($n-1)) {
```

```
    ## print("Padding left edge of chromatin\n");
    $chromatin = pad_left($chromatin, $n - 1 - $n_left_blanks);
  }
  ## ----------- right end ------------
  my $right_i = $chromatin->{'max'};
  my $n_right_blanks = 0;
 CHECK_RIGHT: foreach my $nuci (reverse(($right_i-$n+2)..$right_i)) {
    if (is_blank(get_nucleosome($chromatin, $nuci))) {
      $n_right_blanks++;
    }
    else {
      last CHECK_RIGHT;
    }
  }
  if ($n_right_blanks < ($n-1)) {
    ## print_chromatin($chromatin);
    ## print("\nPadding right edge of chromatin\n");
    $chromatin = pad_right($chromatin, $n - 1 - $n_right_blanks);
    ## print_chromatin($chromatin);
    ## print "\n";
  }
  $chromatin;
}

## updated
sub get_n_nucleosomes {
  my $chr = shift;
  my $location = shift;
  get_nucleosomes($chr, $location, $location + get_n() - 1);
}

sub all_nucleosomes {
  my $chr = shift;
  get_nucleosomes($chr, $chr->{'min'}, $chr->{'max'});
}

sub print_rule_two_lines {
  my $rule = shift;
  my $location = shift;
  my $chromatin = shift;
  my $lhs_string = lhs_string($rule);
  my $rhs_string = rhs_string($rule);
  my $empty_nuc = " " x (get_k() + 1);
  ## write spaces before rule location
  my $offset = $location - $chromatin->{'min'};
  my $spaces = $empty_nuc x $offset;
  print $spaces . $lhs_string . "\n" . $spaces . $rhs_string . "\n";
}

sub print_chromatin {
  my $chr = shift;
  print join(" ", all_nucleosomes($chr));
  ## print "   length: " . chromatin_length($chr) . "\n";
  ## print "   min: " . $chr->{'min'} . "\n";
  ## print "   max: " . $chr->{'max'} . "\n";
  ## foreach my $i (keys %{$chromatin->{'tape'}}) {
  ## print "    $i " . $chromatin->{'tape'}{$i} . "\n";
```

```
  ## }
}

## updated.
sub read_chromatin {
  my $file = shift;
  my $chromatin = {};
  my @nucs = ();
  open (IN, $file) || die("Could not read chromatin file $file");
  while (my $line = <IN>) {
    $line = trim_line($line);
    foreach my $nuc (split /\s/, $line) {
      push(@nucs, $nuc);
    }
  }
  close(IN);
  my $k = check_nucleosome_lengths(@nucs);
  setup_chromatin($k, \@nucs);
}

## updated.
sub setup_chromatin {
  my $k = shift;
  my $nucs = shift;
  my @nucs = @{$nucs};

  if ($k != get_k()) {
    die ("Chromatin nucleosome length does not match nucleosome length in
rules");
  }
##  @blanks = make_blank_pad(get_n()-1);
##  print "*** " . $blanks[0] . "\n";
##  print "*** " . $#blanks . "\n";
  ##
##  print "BEFORE: " . join(" ", @nucs) . "\n";
##  @nucs = (@blanks, @nucs, @blanks);
##  print "AFTER: " . join(" ", @nucs) . "\n";
  my $chromatin;
  foreach my $i (0..$#nucs) {
    $chromatin->{'tape'}{$i} = $nucs[$i];
  }
  ## min and max
  $chromatin->{'min'} = 0;
  $chromatin->{'max'} = $#nucs;
  ## Pad the tape.
  $chromatin = pad_chromatin($chromatin);
  $chromatin;
}

sub blank_nucleosome {
  my $nuc = "";
  foreach my $i (1..get_k()) {
    $nuc = $nuc . "B";
  }
  $nuc;
}
```

```
sub make_blank_pad {
  my $length = shift;
  my $nuc = "";
  foreach my $i (1..get_k()) {
    $nuc = $nuc . "B";
  }
  my @nucs = ();
  ## Only need to add n-1 blank nucleosomes; a rule of all blanks is not
allowed.
  foreach my $j (1..$length) {
    push(@nucs, $nuc);
  }
  @nucs;
}

1;
```

References

1. Luc PV, Tempst P (2004) PINdb: a database of nuclear protein complexes from human and yeast. Bioinformatics 20: 1413-1415.
2. Reece-Hoyes JS, Deplancke B, Shingles J, Grove CA, Hope IA, et al. (2005) A compendium of Caenorhabditis elegans regulatory transcription factors: a resource for mapping transcription regulatory networks. Genome Biol 6: R110.
3. (2004) Finishing the euchromatic sequence of the human genome. Nature 431: 931-945.
4. Field Y, Kaplan N, Fondufe-Mittendorf Y, Moore IK, Sharon E, et al. (2008) Distinct modes of regulation by chromatin encoded through nucleosome positioning signals. PLoS Comput Biol 4: e1000216.
5. Widom J (1998) Structure, dynamics, and function of chromatin in vitro. Annu Rev Biophys Biomol Struct 27: 285-327.
6. Davey CA, Sargent DF, Luger K, Maeder AW, Richmond TJ (2002) Solvent mediated interactions in the structure of the nucleosome core particle at 1.9 a resolution. J Mol Biol 319: 1097-1113.
7. Zamudio NM, Chong S, O'Bryan MK (2008) Epigenetic regulation in male germ cells. Reproduction 136: 131-146.
